# Inactivated Pseudomonas PE(ΔIII) exotoxin fused to neutralizing epitopes of PEDV S proteins produces a specific immune response in mice

**DOI:** 10.1186/s44149-021-00021-9

**Published:** 2021-10-08

**Authors:** Leqiang Sun, Yajie Tang, Keji Yan, Huanchun Chen, Huawei Zhang

**Affiliations:** 1 State Key Laboratory of Agricultural Microbiology, Huazhong Agricultural University, Wuhan, 430070 Hubei China; 2 Laboratory of Animal Virology, College of Veterinary Medicine, Huazhong Agricultural University, Wuhan, 430070 Hubei China; 3 Henan Joint International Research Laboratory of Veterinary Biologics Research and Application, Anyang Institute of Technology, Anyang, 455000 Henan China; 4 College of Life Science and Technology, Huazhong Agricultural University, Wuhan, 430070 Hubei China

**Keywords:** Porcine epidemic diarrhea virus, S protein, Neutralizing antibody, Th1-type immune response

## Abstract

Porcine epidemic diarrhea (PED) caused by the porcine epidemic diarrhea virus (PEDV), is a severe infectious and devastating swine disease that leads to serious economic losses in the swine industry worldwide. An increased number of PED cases caused by variant PEDV have been reported in many countries since 2010. S protein is the main immunogenic protein containing some B-cell epitopes that can induce neutralizing antibodies of PEDV. In this study, the construction, expression and purification of Pseudomonas aeruginosa exotoxin A (PE) without domain III (PEΔIII) as a vector was performed for the delivery of PEDV S-A or S-B. PE(ΔIII) PEDV S-A and PE(ΔIII) PEDV S-B recombinant proteins were confirmed by sodium dodecyl sulfate-polyacrylamide gel electrophoresis and Western blot analysis. The immunogenicity of PEDV S-A and PEDV S-B subunit vaccines were evaluated in mice. The results showed that PEDV-S-B vaccine could not only induce specific humoral and Th1 type-dominant cellular immune responses, but also stimulate PEDV-specific mucosal immune responses in mice. PEDV-S-B subunit vaccine is a novel candidate mucosal vaccine against PEDV infection.

## Main text

Porcine epidemic diarrhea (PED) caused by the porcine epidemic diarrhea virus (PEDV) is a highly contagious and devastating swine disease that leads to high mortality in piglets as it causes severe diarrhea, vomiting and dehydration (Gerdts and Zakhartchouk 2017; Reveles-Félix et al. 2020). PEDV belongs to the Alpha-coronavirus genus of the Coronaviridae family (Lee 2015; Wang et al. 2016; Liu et al. 2019). PEDV genome is a positive-sense and single-stranded RNA of approximately 28 kb in length. It comprises four structural proteins, namely, spike (S), envelope, membrane and nucleocapsid proteins, and three nonstructural proteins, namely, replicases 1a, replicases 1b and ORF 3 (Lee 2015; Sun et al. 2016). Since 2010, PEDV infection outbreaks in pigs have been reported in many Asian countries, including China, South Korea, and Japan (Park et al. 2013; Lee 2015; Park et al. 2018; Pizzurro et al. 2018; Sun et al.^ 2018^; Wang et al. 2018; Liu et al. 2019; Liang et al. 2020; Chen et al. 2021). These outbreaks were caused by a new PEDV strain variant that differs from the classic European strain (CV777). Variant PEDV strains have been reported worldwide since 2010 (Lin et al, 2016; Wang et al. 2016; Guo et al. 2018; Liang et al. 2020; Reveles-Félix et al. 2020). Phylogenetic analysis has revealed that PEDV had two major genetic groups, namely, GI (classical) and GII (variant). GI and GII are further divided into two subgroups (GI-a and GI-b) and three subgroups (GII-a, GII-b, and GII-c) respectively (Guo et al. 2018).

PEDV S glycoprotein is a major immunogenic protein that induces high levels of neutralizing antibodies (Makadiya et al. 2016; Hou et al. 2018; Li et al. 2018a; Li et al. 2018b; Chen et al. 2021). It is also an important target antigen for the development of genetically engineered vaccines against PEDV infection. S protein is approximately 4161 bp long and encodes a protein of approximately 1387 amino acids (aa), which are divided into S1 and S2 subunits (Oh et al. 2014; Makadiya et al. 2016). Multiple neutralizing epitopes (499-638, 748-755, 764-771 and 1368-1374 aa) have been reported in S protein, and they play important roles in humoral immunity (Li et al. 2017; Hao et al. 2017; Liu et al. 2019). Previous studies also suggested that S10 subunit (19-210 aa) can induce neutralizing antibodies against PEDV and mainly neutralizes the infectivity of homologous PEDV strains (Li et al. 2017). Attenuated or inactivated PEDV vaccines based on isolated variant PEDV strains have been successfully developed in several countries (Collin et al. 2015; Lee et al. 2018; Chen et al. 2021). Many researchers have also developed PEDV subunit vaccines based on PEDV S protein (Makadiya et al. 2016; Ma et al. 2018; Li et al. 2018a; Li et al. 2018b).

Pseudomonas aeruginosa exotoxin A (PE) lacking domain III (PE(ΔIII)) is a nontoxic bioadjuvant that enhances cellular and humoral immune responses to a fused target gene (Chen et al. 2001; Liao et al. 2005; Yang et al. 2013). PE contains three domains, and domain III is toxic. As an immune adjuvant, PE(ΔIII) fused with RR1 of Mycoplasma hyopneumoniae enhances specific immune responses (Chen et al. 2001). Chao-Wei Liao et al. (2005) reported that PE(ΔIII), a carrier fused with human papillomavirus type 16 E7, increased the number of E7-specific CD8+ and CD4+ T-cell precursors and improved E7-specific antibodies (Liao et al. 2005). Therefore, PE(ΔIII)-fused PEDV S protein can be used to develop subunit vaccines.

**Fig. 1:**
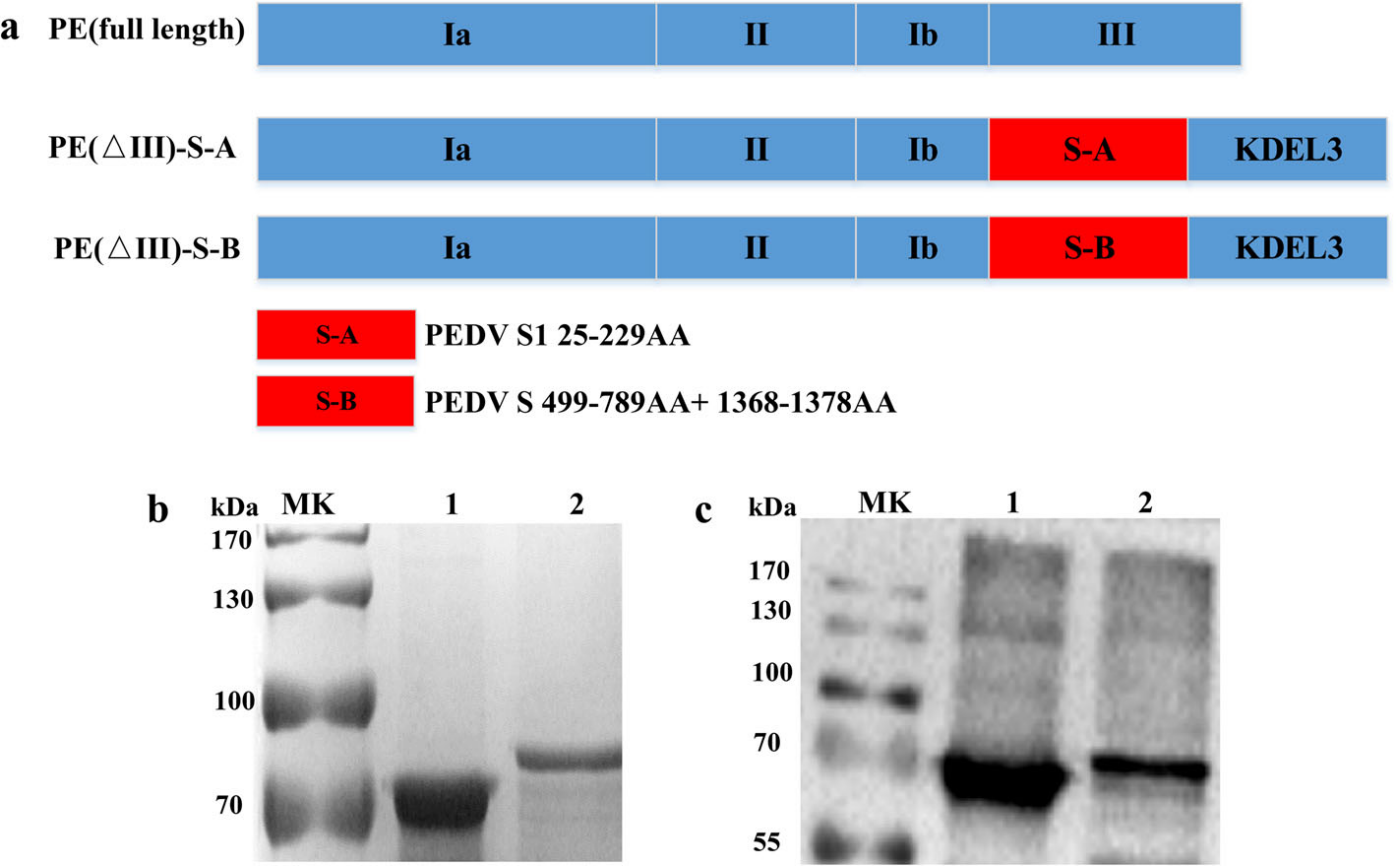
Schematic and expression of the recombinant protein. **a** PE, full-length sequence of PE toxin contains four domains (Ia, II, Ib, III); PE (ΔIII) represents full-length sequence of a PE toxin deletant that deleted domain III. S-A, PEDV S1 aa 25-229; S-B, PEDV S aa 499-789+aa 1370-1378. **b** SDS-PAGE analysis of PE(ΔIII)-PEDV-S-A and PE(ΔIII)-PEDV-S-B proteins purified through affinity chromatography. Lane 1: PE(ΔIII)-PEDV-S-A (71 kDa). Lane 2: PE(ΔIII)-PEDV-S-B (80.5 kDa). **c** Western blot analysis of PE(III)-PEDV-S-A and PE(III)-PEDV-S-B proteins. Lane 1: PE(ΔIII)-PEDV-S-A. Lane 2: PE(ΔIII)-PEDV-S-B. Mouse anti-HisMAb and goat anti-mouse IgG-HRP were used as primary and secondary antibodies, respectively. PE, Pseudomonas aeruginosa exotoxin A; PEDV, porcine epidemic diarrhea virus

In the present study, PE(ΔIII) was constructed, expressed and purified as a vector to deliver PEDV S-A (aa 25–229) or S-B (aa 499–789+ aa 1370–1378) gene segments of the epidemic PEDV isolates of genogroup GII-a. In Fig. 1a, *PEDV S-A* or *S-B* gene was fused with PE(ΔIII) gene *PE(ΔIII) PEDV S-A* or *PE(ΔIII) PEDV S-B*. These two genes were synthesized by Sangon Biotech and cloned into the pET28a vector by restriction enzymes *Nhe*I and *Xho*I. The recombinant plasmids pET28-PE(ΔIII) PEDV S-A and pET28-PE(ΔIII) PEDVS-B were confirmed through restriction enzyme digestion and DNA sequencing.

The recombinant plasmids pET28-PE(ΔIII) PEDV S-A and pET28-PE(ΔIII) PEDVS-B were transformed into *E. coli* BL21 (DE3) pLys cells. Expression of the recombinant strains was induced by 1 mM IPTG and expressed as an inclusion body. After the inclusion bodies were denatured and renatured, the recombinant proteins were purified through Ni^2+^ affinity chromatography. The purified proteins were detected through SDS-PAGE (Fig. 1b) and Western blot analyses (Fig. 1c). SDS-PAGE showed that the purified PE(ΔIII) PEDV S-A and PE(ΔIII) PEDVS-B were approximately 71 and 80.5 kDa, respectively. The specific bands were determined by His monoclonal antibody through Western blot.

**Fig. 2:**
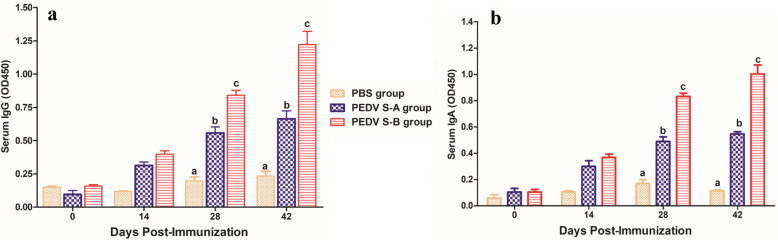
PEDV-specific IgG and IgA in the sera of the immunized mice by indirect ELISA. **a** PEDV-specific IgG detected by indirect ELISA. **b** PEDV-specific IgA detected by indirect ELISA. Data were represented as the mean ± SEM. Different letters (**a**, **b**) indicate a statistically significant difference between the different experimental groups (*P* < 0.05). PEDV, porcine epidemic diarrhea virus

**Fig. 3:**
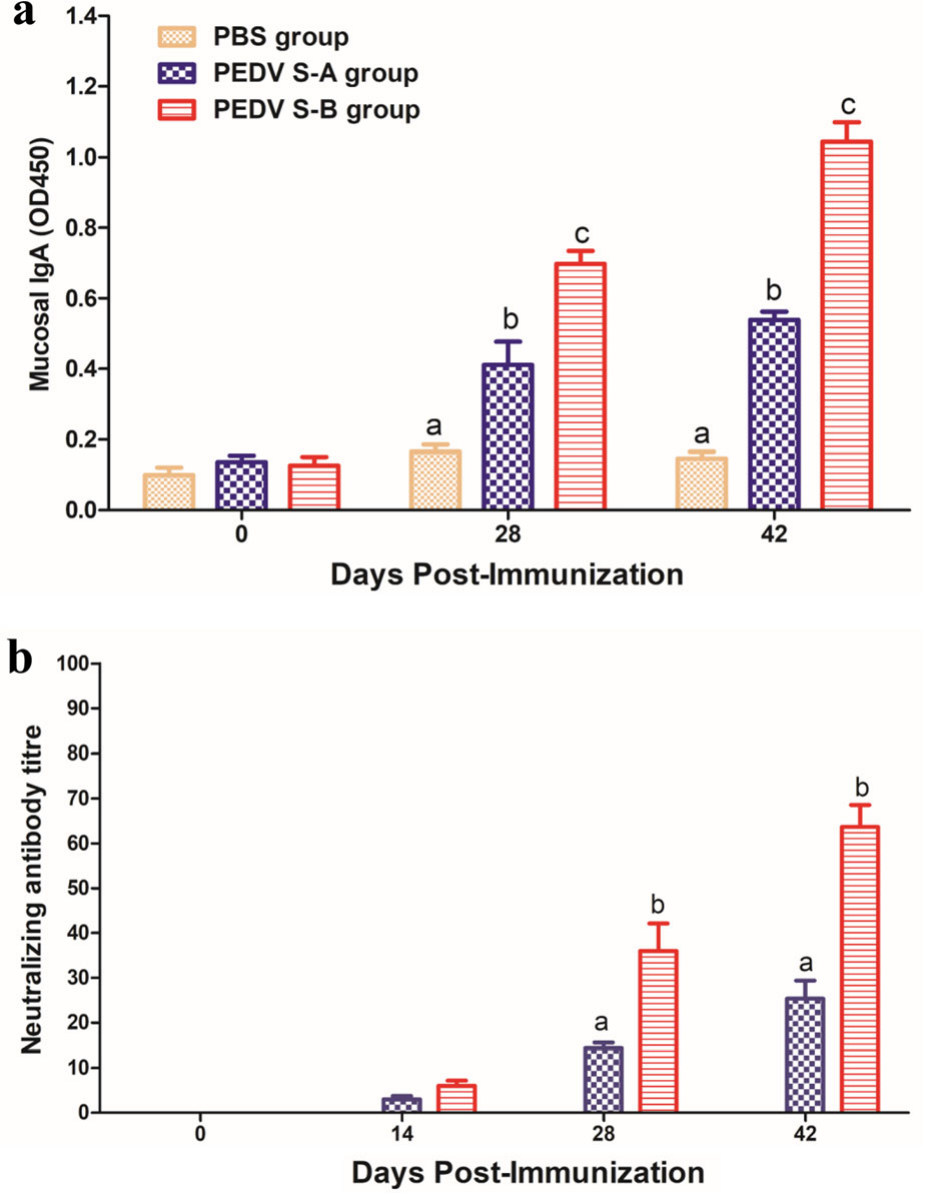
Detection of PEDV-specific IgA in the small intestines of mice and PEDV-specific neutralizing antibodies. **a** Mucosal IgA detection. **b** Serum neutralizing antibody titers against PEDV. Data are represented as the mean ± SEM. Different letters (**a**, **b**) indicate a statistically significant difference between the different experimental groups (*P* < 0.05). PEDV, porcine epidemic diarrhea virus

To evaluate the specific humoral immunity of mice immunized with the recombinant proteins, indirect ELISA and neutralizing antibody tests were performed. Serum samples collected at 0, 14, 28, and 42 dpi (days post immunization) were examined by indirect ELISA. As showen in Fig. 2a, PEDV-specific IgG could be detected in mice immunized with PEDV S-A and PEDV S-B at 14 dpi. After mice were boosted with the vaccine, antibody titers of PEDV S-B group were significantly higher than those of PEDV S-A group (*P *> 0.05). In Fig. 2b, PEDV-specific IgA was detected in mice immunized with PEDV S-A and PEDV S-B at 28 and 42 dpi. PEDV-specific IgA level of PEDV S-B group was significantly higher than that of PEDV S-A group. In addition, PEDV-specific IgA in the intestinal contents of mice immunized with PEDV S-A or PEDV S-B was detected (Fig. 3a). IgA level of mice immunized with PEDV S-B was significantly higher than that of PEDV S-A group. Specific PEDV IgAs play an important role in PEDV vaccines. PEDV-specific neutralizing antibody could also be stimulated by immunization with PEDV S-A or PEDV S-B vaccine. The levels of neutralizing antibodies in PEDV S-B group were higher than those in PEDV S-A group (Fig. 3b). Specific IgA and neutralizing antibodies are important factors for providing protection against PEDV.

**Fig. 4:**
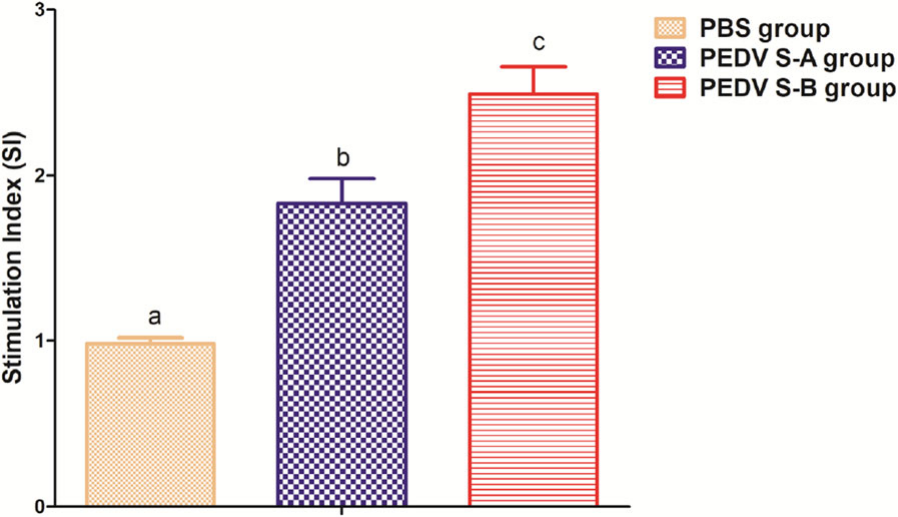
Lymphocyte proliferation responses measured by MTS assay in mice. Data were represented as the mean ± SEM. Different letters (**a**, **b**) indicate a statistically significant difference between different experimental groups (*P* < 0.05). PEDV, porcine epidemic diarrhea virusPEDV, porcine epidemic diarrhea virus

**Fig. 5:**
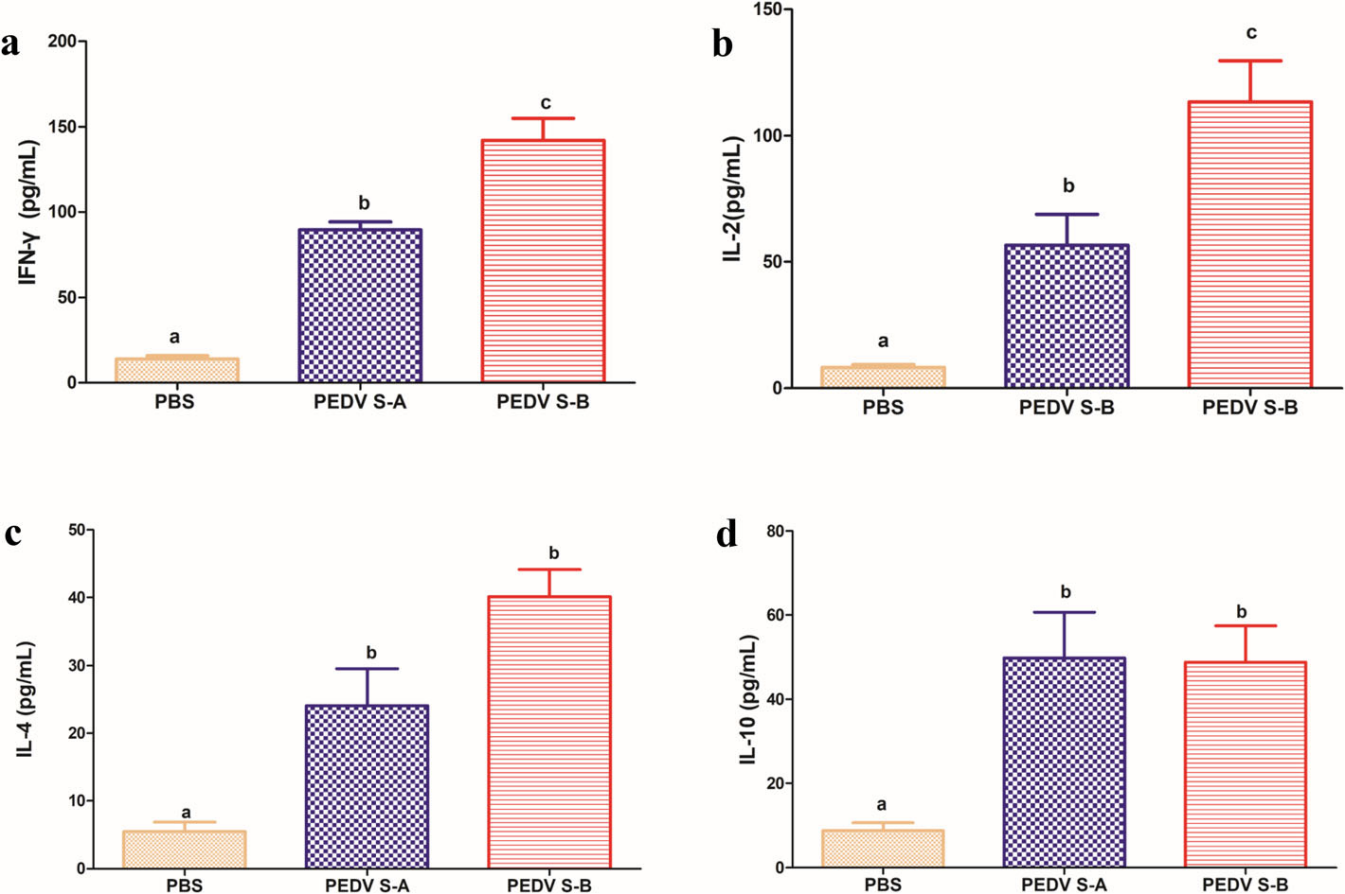
Cytokine levels in the culture supernatants. IFN-γ (**a**), IL-2 (**b**), IL-4 (**c**), and IL-10 (**d**) levels were measured by double-antibody sandwich ELISA. Data were represented as the mean ± SEM. Different letters (**a**, **b**) indicate a statistically significant difference between different experimental groups (*P* < 0.05). PEDV, porcine epidemic diarrhea virusPEDV, porcine epidemic diarrhea virus

To characterize cell-mediated immune responses, PEDV-specific lymphocyte proliferation and cytokine profiles were determined. Proliferative responses of the lymphocytes stimulated by purified PEDV were observed in PEDV S-A and PEDV S-B groups but not in PBS group (Fig. 4). The stimulation index (SI) of PEDV S-A significantly differed among PEDV S-B groups. These results indicated that PEDV S-A and PEDV S-B could induce the proliferation of spleen lymphocytes *in vitro*. Th1-type (IFN-γ and IL-2) and Th2-type (IL-4 and IL-10) cytokines were examined using a commercially available double-antibody sandwich ELISA kit. As showen in Fig. 5a and b, the highest concentrations of IFN-γ and IL-2 were measured in PEDV S-B group (*P* < 0.001), and they were significantly higher than those of PEDV S-A group. In Fig. 5c and d, concentrations of IL-4 and IL-10 in PEDV S-A and PEDV S-B groups were significantly higher than those in PBS group. No significant difference was observed between PEDV S-A and PEDV S-B groups (*P* > 0.05). Cytokines play an important regulatory role in various immune reactions. These results demonstrated that PEDV S-B group could induce Th1-dominant cellular immune responses. Our results were consistent with previous studies, which showed that PE(ΔIII) delivering antigen proteins improved cellular immune responses *in vivo* (Yang et al. 2013; Martínez et al. 2021).

Based on the above results, the purified PEDV S-A and PEDV S-B proteins could induce specific humoral and cellular immune responses in mice. PEDV-S-B could stimulate PEDV-specific immune responses in mice. This study indicates that PEDV-S-B subunit vaccine is a novel candidate subunit vaccine against PEDV infection. Further studies should be conducted to evaluate the immunoprotective effect of PEDV-S-B subunit vaccines in sows and piglets.

## Methods

### Cells and viruses

Vero cells (ATCC CCL-81) were cultured in Dulbecco’s minimal essential medium (DMEM; Gibco, USA) with 10% fetal bovine serum (FBS; Gibco, USA) at 37 °C in 5% CO_2_. PEDV CH2019 strain, which is the pandemic strain of genogroup GII-a, was isolated and stored in our laboratory. PEDV CH2019 strain was propagated in Vero cells and used in the neutralization assay.

### Construction of plasmids and protein expression and purification

PEDV S-A fused with PE(ΔIII) (GenBank: K01397.1) or PEDV S-B from the variant PEDV CH2019 strain was codon optimized, synthesized by Sangon Biotech, and cloned into pET28a vector for recombinant protein expression. Two recombinant plasmids, namely, pET28-PE(ΔIII) PEDV S-A and pET28-PE(ΔIII) PEDV S-B, were successfully constructed and confirmed through DNA sequencing by Sangon Biotech.

The recombinant plasmids pET28-PE(ΔIII) PEDV S-A and pET28-PE(ΔIII) PEDV S-B were transformed into *E. coli* BL21 (DE3) pLys cells, and the recombinant proteins were purified by Ni^2+^ affinity chromatography.

### Sodium dodecyl sulfate-polyacrylamide gel electrophoresis (SDS–PAGE) and Western blot assays

After purification was performed, the proteins (PE(ΔIII) PEDV S-A and PE(ΔIII) PEDV S-B) were separated through 12% SDS-PAGE to detect the purity of target proteins. The purified target proteins were also confirmed by Western blot analysis. Protein bands were detected using an ECL chemiluminescence system and visualized with Image Lab V. 4.0.1.

### Mice immunization

Animal experiments were performed in accordance with protocols approved by the animal ethical and welfare committee of Huazhong Agricultural University. Six-week-old female BALB/c mice were purchased from the Hubei Center of Disease Control, China. The mice were randomly divided into three groups (*n* = 20). Group A (negative control group) was intranasally (i.n.) immunized with 50 μL PBS; Group B was i.n. immunized with PEDV S-A, which was composed of 30 μg purified PEΔIII PEDV S-A; Group C was i.n. immunized with PEDV S-B, which was composed of 30 μg purified PE(ΔIII) PEDV S-B. After 2 weeks, all mice were boosted with the same vaccine. Serum samples were collected at 0, 14, 28, and 42 dpi and assayed for PEDV-specific IgG or IgA and PEDV-specific neutralizing antibodies. The small intestines of mice immunized with PEDV S-A and PEDV S-B groups were collected at 42 dpi. The extracted liquid was harvested by grinding and centrifuging, and it was assayed for PEDV-specific IgA.

### Enzyme-linked immunosorbent assay

Ninety-six well microtitration plates were coated with 0.5 μg purified PEDV diluted in 0.05 M NaHCO_3_ with a sucrose discontinuous gradient and incubated at 4 °C overnight. The incubated plates were blocked with 2% BSA-PBST and washed three times with PBST. Samples were diluted (1: 200) with 2% BSA-PBST, added to the plates for 1 h at 37 °C, washed three times, and incubated with HRP-conjugated goat anti-mouse IgG (1: 5000; ABclonal, USA) or goat anti-mouse IgA (1: 4000; Southern Biotech, USA) for 1 h at 37 °C to detect antibody titers of PEDV-specific IgG or IgA. The optical density (OD) was measured at 450 nm by using a microplate reader.

### Neutralization assay

Serum neutralization test was performed to detect PEDV antibodies as previously described (Hou et al. 2018; Li et al. 2018a; Li et al. 2018b). Neutralizing antibody titers were calculated as the reciprocal of the highest dilution at which Vero cell infection was inhibited in 50% of the culture wells.

### Lymphocyte proliferation assay

Splenic lymphocytes were separated from the spleen of immunized mice by using a mouse lymphocyte isolation reagent (TBD, Tianjin, China). The isolated lymphocytes were adjusted to 4×10^6^ cells/mL, resuspended in RPMI-1640 containing 10% FBS, seeded into 96-well plates (100 μL/well), and stimulated with 5 μg purified PEDV, 10 μg/mL concanavalin A, or 100 μL RPMI 1640. Proliferative responses were detected with an MTS assay. OD was measured at 450 nm by using a microtiter plate reader. Stimulation index (SI) was calculated with the following formula: SI = (OD of immunized groups – OD of blank control)/(OD of negative control group – OD of blank control).

### Cytokine assays

Splenic lymphocytes were isolated, resuspended at 4×10^6^ cells/mL with RPMI-1640 containing 10% FBS, seeded into 24-well plates, and stimulated with 5 μg/mL purified PEDV. After 24 h of incubation, culture supernatant was centrifuged at 1000 rpm for 10 min. Cytokines (IFN-γ, IL-2, IL-4, and IL-10) in the culture supernatant were analyzed with a commercial sandwich ELISA kit. OD was measured at 450 nm by using a microtiter plate reader.

### Statistical analyses

Statistical analyses were performed with GraphPad Prism V. 5. Data were represented as the mean ± SEM. One-way ANOVA was conducted to statistically analyze differences between multiple groups. Differences were considered significant when *P* < 0.05.

## Data Availability

Data will be shared upon request by the readers.

## Abbreviations

PED: Porcine epidemic diarrhea
PEDV: Porcine epidemic diarrhea virus
PE: Pseudomonas aeruginosa exotoxin A
Th1: T helper (Th1) cells
E. coli: Escherichia coli
kDa: Kilodalton
ELISA: Enzyme-linked immunosorbent assay
IgA: Immunoglobulin A
SI: Stimulation index
